# The role of vector control in stopping the transmission of malaria: threats and opportunities

**DOI:** 10.1098/rstb.2013.0431

**Published:** 2014-06-19

**Authors:** Janet Hemingway

**Affiliations:** Liverpool School of Tropical Medicine, Pembroke Place, Liverpool L3 5QA, UK

**Keywords:** pyrethroid, resistance, insecticide, mosquito

## Abstract

Malaria control, and that of other insect borne diseases such as dengue, is heavily dependent on our ability to control the mosquito populations that transmit these diseases. The major push over the last decade to reduce the global burden of malaria has been driven by the distribution of pyrethroid insecticide-treated bednets and an increase in coverage of indoor residual spraying (IRS). This has reduced malaria deaths by a third. Progress towards the goal of reducing this further is threatened by lack of funding and the selection of drug and insecticide resistance. When malaria control was initially scaled up, there was little pyrethroid resistance in the major vectors, today there is no country in Africa where the vectors remain fully susceptible to pyrethroids. The first pyrethroid resistance mechanisms to be selected produced low-level resistance which had little or no operational significance. More recently, metabolically based resistance has been selected, primarily in West Africa, which in some mosquito populations produces more than 1000-fold resistance. As this spreads the effectiveness of pyrethroid-based bednets and IRS will be compromised. New public health insecticides are not readily available. The pipeline of agrochemical insecticides that can be re-purposed for public health dried up 30 years ago when the target product profile for agricultural insecticides shifted from broad spectrum, stable, contact-acting insecticides to narrow spectrum stomach poisons that could be delivered through the plant. A public–private partnership, the Innovative Vector Control Consortium, was established in 2005 to stimulate the development of new public health pesticides. Nine potential new classes of chemistry are in the pipeline, with the intention of developing three into new insecticides. While this has been successfully achieved, it will still take 6–9 years for new insecticides to reach the market. Careful management of the resistance situation in the interim will be needed if current gains in malaria control are not to be reversed.

## 1. Introduction

Malaria is still a major health issue in much of the world with around 660 000 deaths and 219 million cases still occurring in 2010 [1]. This represents a major improvement over the past decade with a 33% reduction in malaria deaths in Africa, where 90% of the world's malaria deaths occur, with massive scaling up of disease prevention activities linked to attempting to fulfil the Millennium Development Goals (MDGs) of reduced maternal and child mortality. Malaria features as a specific indicator for MDG 6 and also contributes to other MDGs, including poverty, child survival, maternal health and education. The majority of malaria prevention activities centre around controlling the mosquito vectors by indoor residual spraying (IRS) of houses with long-lasting insecticide formulations, or reducing the ability of the insects to bite people by encouraging them to sleep under long-lasting insecticide-treated bednets (LLINs).

The World Health Organisation (WHO) estimates that in the decade 2000–2010, 274 million cases of malaria and 1.1 million deaths, the majority in children under 5 years of age in sub-Saharan Africa, were prevented by these activities. Over this period, LLIN use rose from 3% in 2000 to 53% in 2011, where it reached a plateau. To achieve universal coverage with LLINs, 780 million people at risk of malaria would need to have access in Africa, and 150 million bed nets would need to be delivered each year. This calculation assumes that nets remain effective on average for 5 years. In 2012, this target was not met with only 66 million nets being distributed. There is also a growing body of evidence which suggests that the LLINs are unlikely to last for 5 years [2,3]. The LLIN market is very price sensitive, and cheaper nets with lower tensile strength have dominated the market, resulting in nets that tear and become unusable well before the projected 5 year life expectancy is reached.

IRS rose from 5% in 2005 to 11% in 2011, representing 153 million people protected globally, 77 million of these in Africa [1]. The increase was largely due to the efforts of the President's Malaria Initiative (PMI) in 19 focus African countries, with 30.3 million people protected by IRS in 2012 [4].

If we are to maintain these improvements in disease control and push towards regional elimination and eventual global eradication of malaria, then funding levels to support control efforts and operational activities need to be increased substantially. In order to reach the required global malaria targets, an estimated US$5.1 billion will be needed every year from 2011 to 2020. In 2011, only US$2.3 billion was available, less than half the figure needed. This has improved recently with increased commitments in December 2013, with US$12 billion pledged against the US$ 15 billion needed for 2014–2016 for the Global Fund [5]. However, an increase in financial support alone will not be enough. We need to exert better stewardship of the insecticides and drugs needed to prevent and treat the disease, to ensure that they remain viable in the medium to long term. If we do not do this now, then resistance will inevitably reduce our ability to prevent and control malaria.

## 2. Resistance management

Resistance to both drugs and insecticides is becoming a major issue. The epicentre of drug resistance is in Southeast Asia, where resistance to the first line artemisinin combination therapy (ACT) treatments was selected in Cambodia and has since spread to neighbouring countries [6]. Monitoring this resistance is not simple as there are currently no easy markers that can be tracked. ACT resistance is suspected when an increase in parasite clearance times is observed and more than 10% of malaria cases still have detectable parasitaemia 3 days after ACT treatment. The threat this resistance poses to our ability to treat malaria resulted in the WHO establishing the Global Plan for Artemisinin Containment (GPARC) in 2011 [7]. While this resistance is a growing issue in Asia, it has yet to be detected as a major issue in Africa.

By contrast, insecticide resistance is already a major issue in Africa. In Africa, where the greatest burden of malaria mortality occurs, there are two major malaria vectors, *Anopheles gambiae* and *An. funestus*, although several secondary vectors occur, which can take on a primary role. *An. gambiae* M and S forms have now been recognized as separate species, *An. gambiae* (S form) and *An. coluzzi* (M form) [8]. Throughout much of sub-Saharan Africa, these vectors occur [9], although their importance may shift temporally [10]. *An. gambiae* breeds in small temporary water bodies and semi-permanent sites without vegetation, and densities fluctuate, increasing in the rainy seasons which produce abundant breeding sites, whereas *An. funestus* uses larger bodies of permanent clean water that are fringed with vegetation. *An. gambiae* is much easier to colonize and handle in the laboratory, and because of this, and the historical ease with which *An. funestus* was controlled with DDT in the 1960s, *An. funestus* was wrongly felt by many to be a less important vector.

Current practices in malaria prevention are a classic example of poor product stewardship likely to lead to the rapid selection and spread of resistance. In many ways, the pyrethroids are ideal insecticides for preventing biting and controlling mosquitoes. They act rapidly, many pyrethroids have both a repellent and a killing function, they are relatively safe for use in close proximity to humans, and they are easy to formulate and relatively cheap to produce. No other insecticide class combines this set of desirable characteristics. This makes them the insecticide class of choice for disease prevention. Today, all LLINs and more than 80% of IRS campaigns use pyrethroids. This represents a major selection pressure for pyrethroid resistance, with the inevitable result that we have moved from pyrethroid resistance being a rare occurrence in sub-Saharan African malaria vectors in 2000 to the present time, when no African country has fully pyrethroid susceptible malaria vectors. Resistance is now widespread in both *An. gambiae* and *An. funestus*, but the intensity and diversity of the resistance selected vary with location and species [11,12].

The extent of selection and spread of pyrethroid resistance has now prompted the WHO to publish the Global Plan for Insecticide Resistance Management (GPIRM) [13], which is a concerted call to action and aims to provide guidance to countries on how to develop and implement an insecticide resistance management plan within their operational malaria control activities.

Pyrethroid resistance has occurred in waves and we have been fortunate that the resistance selected initially has been low level and has had little obvious impact on the effectiveness of pyrethroid-based IRS or LLINs. This situation is now changing rapidly. The initial wave of pyrethroid resistance was primarily a re-selection of an old DDT resistance mechanism in *An. gambiae*. DDT and pyrethroids have a common mode of action, binding to the sodium channels on the nerve membranes. Two common mutations on the sodium channel involving a leucine residue being converted to phenylalanine or serine produce the common ‘*kdr*’ phenotypes, with insects being less susceptible to the insecticide and more difficult to rapidly knock down after pyrethroid exposure. Often referred to as the West or East African forms of *kdr* in *An. gambiae*, indicating the locations in which the respective mutations were first detected, these mutations have actually arisen multiple times and spread from different nodes [14], becoming the dominant phenotype in *An. gambiae* in many parts of Africa. The mutations produced by a simple base change can easily be detected using simple PCR. The ease of monitoring in this form has resulted in several groups using this as a surrogate for comprehensive resistance monitoring, with the potential for under-reporting of true resistance levels. *Kdr*, although common in many insect species, has not been selected to date in *An. funestus*.

There is no convincing evidence that *kdr* alone produces operationally significant levels of pyrethroid resistance. This is in direct contrast to the situation in houseflies, where selection of *kdr* and subsequently *super-kdr* decimated the market for pyrethroid-based housefly control products [15]. Experimental hut studies with LLINs suggested little or no impact of *kdr* on the ability of the resistant mosquitoes to take a blood meal. Similarly, the national malaria control programme in Equatorial Guinea were able to re-instate pyrethoid-based IRS in 2013, despite a high frequency of *kdr* in *An. gambiae*, as part of their evidence-based insecticide resistance management plan [16].

The second wave of resistance selection produced multiple origins of metabolically based mechanisms of pyrethroid resistance in both major vectors. The first major reported instance of this was in *An. funestus* in Mozambique, where a cytochrome P450-based mechanism was detected in 1999 in Kwazulu Natal province [17]. This resistance also occurs in neighbouring Mozambique [18] and was recently detected in *An. funestus* in Malawi [19]. It produces an order of magnitude higher resistance to pyrethroids than *kdr*, with a low level of cross-resistance to the carbamate insecticide, bendiocarb [17]. It is unclear whether this resistance had a direct impact on disease control, as although its selection coincided with a major resurgence of malaria in South Africa, resistance to first line sulfadoxine–pyrimethamine drug treatment was also detected at about the same time and the relative contributions of both resistances are impossible to disentangle. Operationally, however this produced a change in insecticide use policy, with the national programmes in South Africa, Mozambique and Swaziland shifting to bendiocarb or DDT to counter the pyrethroid resistance [20].

In *An. Gambiae*, the first documented metabolic pyrethroid resistance in East Africa was relatively low level [21], but was followed soon after by reports of higher level metabolic resistance in West Africa, commensurate with that seen in South African *An. funestus*. In all cases, these resistances were linked to upregulation of cytochrome P450s which were subsequently shown to metabolize pyrethroids [22]. However, as these metabolic enzymes sit within very large multi-gene families that are part of the normal cellular machinery for detoxifying xenobiotics, and tend to be linked in regulatory pathways to a broad range of other genes which can be up- or downregulated in a coordinated manner, isolating the specific mutations producing the resistance has been problematic. Resistance can be tracked by monitoring the upregulation using qRTPCR, but is not simply tracked using PCR. The difficulty for field-based entomologists tracking this resistance has resulted in major under-reporting of the extent and spread of this type of resistance. It has also made it impossible to draw conclusions from a systematic review of the impact of pyrethroid resistance on LLIN efficacy, where there are no data available to track the potential impact of resistance on disease transmission, and even the simpler relationship between resistance and entomological indicators (such as mortality, repellency and blood feeding), which is apparent in laboratory studies where resistance can be better characterized, is lost in the noise in the data connected to inadequate and inaccurate resistance monitoring.

In 2013, a third wave of P450-based metabolic resistance became apparent in West African *An. gambiae*. This resistance, now recorded in Burkina Faso and the Ivory Coast, produces resistance to pyrethroids an order of magnitude higher again than that manifest in the more widespread metabolic or metabolic + *kdr* populations. Resistance once selected often spreads rapidly, as seen by the rapid shifts of insecticide susceptibility throughout Africa over the past decade and the switch in countries like Malawi from complete susceptibility to country wide resistance over the space of less than 12 months. The levels of resistance (more than 1000-fold) conferred by this third resistance wave give a high probability that this will impact on the efficacy of both pyrethroid-based bednets and IRS.

In addition to physiological resistance, there is the potential for behavioural changes in the insect vectors. This is much more poorly documented, but shifts from indoor biting and resting to outdoors may be occurring in parts of Africa.

## 3. New interventions on the horizon

Development of new drugs, insecticides and diagnostics will be needed if we are to maintain current gains and reduce global burdens of transmission. The risk/reward profiles of these products are not sufficiently balanced for industry to embark on this development alone. The estimated size of the market for crop protection insecticides is approximately US$ 8 billion per annum, non-crop insecticides are approximately US$ 2 billion and vector control is only US$0.2–0.7 billion. The profit margin in this size of market is not sufficient to warrant the risk associated with the US$ 1–200 million cost of developing a new insecticide. Product Development Partnerships (PDPs) have been established to combine public money with public and private know-how to produce products applicable for use in disease endemic countries. Four PDPs are active in malaria, the Medicines for Malaria Venture stimulating drug development, Malaria Vaccine Initiative for vaccines, the Innovative Vector Control Consortium (IVCC) for insecticides and vector control diagnostics and the Foundation for Innovative New Diagnostics for clinical diagnostics.

**Figure 1.**
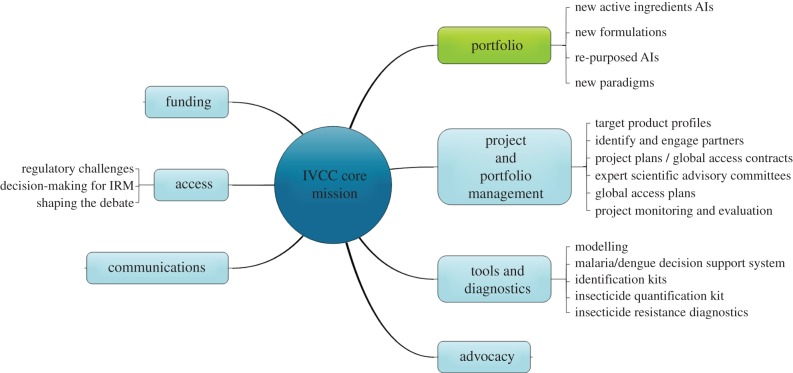
Structure and function of the IVCC. (Online version in colour.)

Pyrethroids were the last mainstream public health insecticide class to be developed, and these have been in use now for more than 30 years. The primary focus of the IVCC is to work with industry to develop new insecticide classes to replace the pyrethroids for both IRS and LLINs [23]. In addition to its portfolio of new insecticide-based products, it also has a number of products designed to improve insecticide resistance monitoring and evaluation, quality assurance of insecticide-based products in field settings and data systems to draw together entomological, parasitological, logistics and disease transmission data (figure 1).

**Figure 2.**
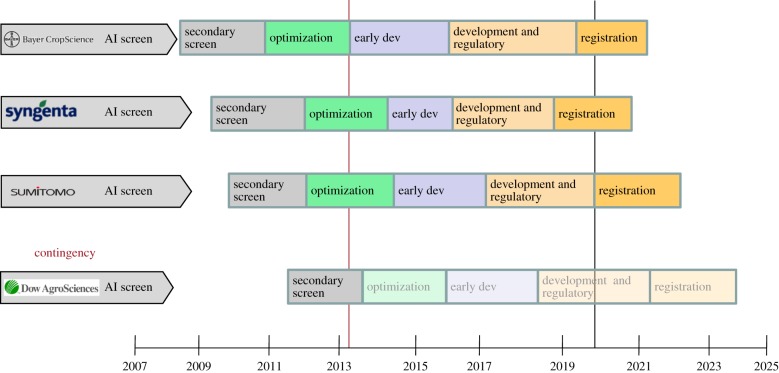
Overview of the new insecticides portfolio of IVCC products and associated timeframes to get these insecticides to the malaria control market. (Online version in colour.)

To ensure that we can maintain control in the medium to long term and establish evidence-based insecticide resistance management plans, more than a single new insecticide class will be required, as we already have resistance issues with all existing public health insecticide classes. Ideally, any new insecticide should not be introduced as a single treatment, resulting in a high resistance selection pressure. Introducing several insecticides in rotation, mosaic or mixture formats should reduce the likelihood of resistance selection significantly, as long as none of the insecticides share a common target site or metabolic detoxification pathway. The current portfolio of IVCC projects is designed to produce three new insecticide classes by around 2023, with no cross-resistance to current insecticide classes. A range of projects have been developed with industrial partners that has resulted in more than 4.5 million compounds being screened in simple mosquito mortality tests, with the most promising leads taken forward into further development. An overview of the current portfolio and projected timelines is given in figure 2.

It is essential that we attempt to truncate this development time given the rapid rise and spread of pyrethroid resistance, but in the interim it is essential that we attempt to manage the current pyrethroid, organophosphate and carbamate resistance circulating in the anopheline populations to maintain control with both LLINs and IRS. This will require improved monitoring and evaluation of resistance in the field aligned with evidence-based resistance management strategies, building on an understanding of local mosquito resistance frequencies, level and operational impact. Current monitoring activities associated with almost all programmes are inadequate for this in both scale and quality. For example, resistance monitoring that relied entirely on spot bioassays using single ‘discriminating dosages’ would have picked up the pyrethroid resistance in West Africa, but would not have registered the shift from 5- to 100- to 1000-fold resistance conferred by the three different waves of resistance that have occurred in countries such as Ivory Coast. Reliance on *kdr* PCR monitoring as a proxy for pyrethroid resistance would have registered the first wave of resistance, but suggested over time that potentially resistance frequencies were declining as resistance due to the metabolic systems increased. There is clearly a need for better informed, more systematic resistance monitoring with data shared openly so a coordinated response to the resistance threat can be established. To date, there is little evidence that this will be achieved in the near future despite the obvious and increasing threat of resistance.

Regulatory and registration times for the new insecticides are at present estimates. All current public health insecticides have been introduced from re-purposing of mainstream agrochemicals that have followed standard regulatory pathways to market. These new insecticides will be the first that have been developed primarily for the public health market, hence there is no internationally agreed regulatory pathway for these insecticides. Appropriate regulatory bodies such as the Environmental Protection Agency and normative bodies such as the World Health Organisation Pesticide Evaluation Scheme are working with the IVCC and other interested parties to look at the appropriate route to market for these compounds. It is likely that international pressure will be needed to ensure that we have a safe, effective and timely regulatory and registration system in place to ensure that these compounds can be used for disease control in a timeframe that will allow replacement of pyrethroids before the major malaria reductions we see today are lost.
